# Supplementary material for: Combined Action of Hyper-Harmonized Hydroxylated Fullerene Water Complex and Hyperpolarized Light Leads to Melanoma Cell Reprogramming In Vitro
Source: Nanomaterials (Basel). 2022 Apr 13;12(8):1331. doi: 10.3390/nano12081331 (PMC9033139; doi:10.3390/nano12081331)
Supplement: Supplementary file 1 [file nanomaterials-12-01331-s001.zip › nanomaterials-1638389-supplementary.pdf]

Supporting information

Combined Action of Hyper-Harmonized Hydroxylated Fullerene Water Complex and Hyperpolarized Light Leads to Melanoma Cell Reprogramming In Vitro

Milica Markelić [1,*], Dijana Drača [2], Tamara Krajnović [2], Zorana Jović [3], Milica Vuksanović [4], Djuro Koruga [3], Sanja Mijatović [2] and Danijela Maksimović-Ivanić [2,*]

[1] Faculty of Biology, University of Belgrade, 11000 Belgrade, Serbia
[2] Institute for Biological Research "Siniša Stanković", National Institute of Republic of Serbia, University of Belgrade, 11060 Belgrade, Serbia; dracadiana@gmail.com (D.D.); tamara_krajnovic@yahoo.com (T.K.); sanjamama@ibiss.bg.ac.rs (S.M.)
[3] TFT Nano Center, 11050 Belgrade, Serbia; zorana.jovic@tftnanocenter.rs (Z.J.); djuro.koruga@gmail.com (D.K.)
[4] Zepto Hyper Tech, 11070 Belgrade, Serbia; milica.vuksanovic@zepterhypertech.com
* Correspondence: milica.markelic@bio.bg.ac.rs (M.M.); nelamax@ibiss.bg.ac.rs (D.M.-I.)

List of contents

Figure S1. UV-Vis-NIR and FTIR absorption spectra of 3HFWC substance before and after irradiation with hyperpolarized light (HPL) for 5, 10 or 15 min

Table S1. Absorbance intensity (a.u) in UV–VIS-NIR domain (200-900 nm) of 3HFWC substance: before, after 5 min, after 10 min and 15 min irradiation with hyperpolarized light (HPL)

Figure S2. Effect of 3HFWC on melanoma cell viability *in vitro*

Figure S3. Effect of HPL on melanoma cell viability *in vitro*

Figure S4. Viability of peritoneal exudate cells (PECs) upon exposure to 3HFWC and/or HPL *in vitro*

Figure S5. Analysis of cell death induction of melanoma cells treated with 3HFWC and/or HPL

Figure S6. Effect of prolonged exposure to 3HFWC/HPL on B16 melanoma cells viability

Figure S7. Immunocytochemical analysis of MBP, a Schwann cells marker in B16 melanoma cells treated with 3HFWC and/or HPL

Figure S8. Endocytotic activity of B16-F10 melanoma cells treated with 3HFWC

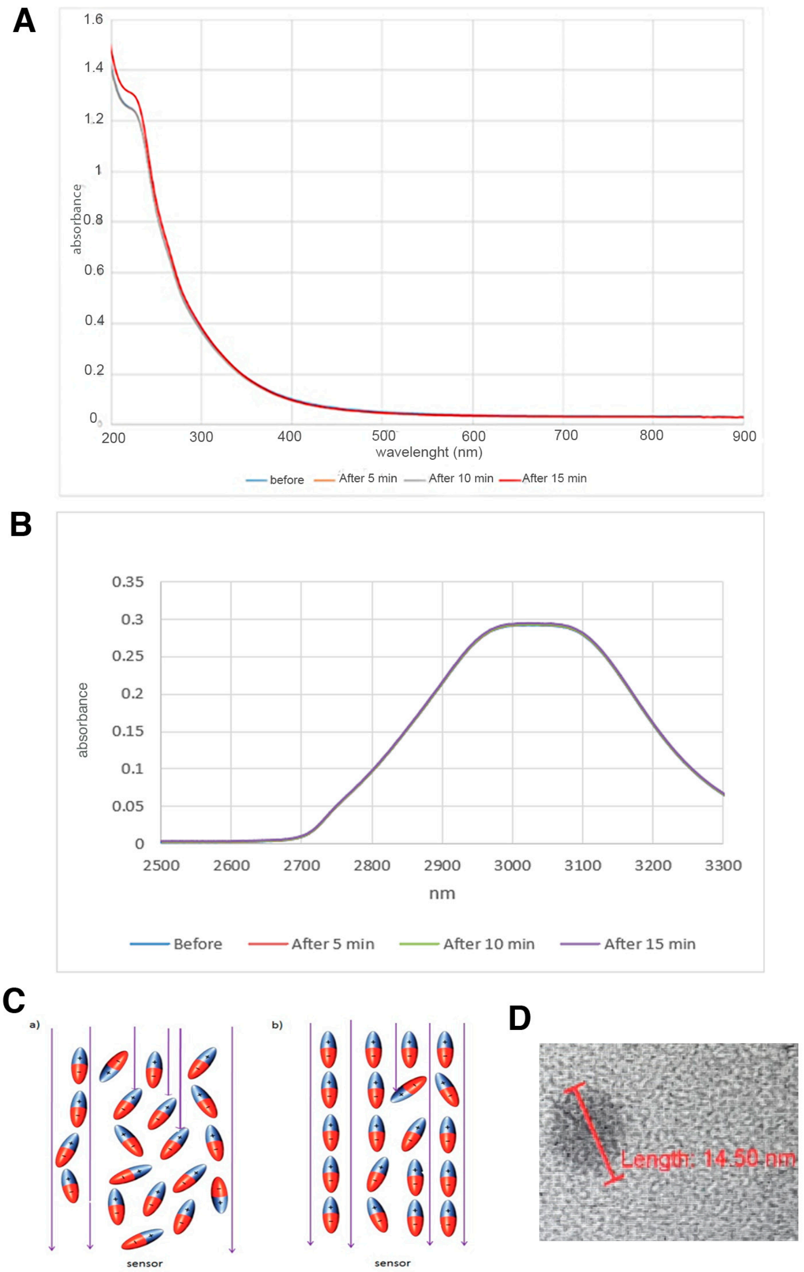

Figure S1. UV-Vis-NIR and FTIR absorption spectra of 3HFWC substance before and after irradiation with hyperpolarized light (HPL) for 5, 10 or 15 min. (**A**) UV-VIS-NIR (200-900 nm) and (**B**) FTIR domain (2500-3300 nm), there is not difference in hydrogen bonds domain (2700-3330 nm). (**C**) Water molecules possess dipole moment (1.85 D or $6.17\times10-30$ C·m) and their orientation is proposed to be in different direction: a) before, and b) after 5, 10 and 15 minutes HPL irradiation. For 5 min and 10 min irradiation there is not difference in absorption spectra in UV-Vis-NIR domain. Only after 15 min irradiation, with HPL, there is small difference in

absorption spectra in 200-300 nm domain (maximal difference is Δ= 0.07 a.u. at 220 nm wavelength). Photons pass through ordering water molecules (3HFWC before irradiation, 5 minutes and 10 minutes after irradiation) smoothly, while in less ordering water molecules (15 minutes after irradiation there is light influence on water molecules in domain 200-300 nm) photons interact with them and absorbed photons. (**D**) 3HFWC solid phase particle size measured by transmission electron microscopy (TEM).

Table S1. Absorbance intensity (a.u) in UV–VIS-NIR domain (200-900 nm) of 3HFWC substance: before, after 5 min, after 10 min and 15 min irradiation with hyperpolarized light (HPL).

Absorbance	200 nm	220 nm	240 nm	260 nm	280 nm	300 nm	300-900 nm
Before irradiation	1.42	1.24	1.03	0.70	0.48	0.37	There is no difference
After 5 min HPL	1.41	1,24	1.03	0.70	0.48	0.37	
After 10 min HPL	1.41	1.24	1.03	0.70	0.48	0.37	
After 15 min HPL	1.48	1.31	1.09	0.74	0.51	0.38	
Difference (Δ)	0.06	0.07	0.06	0.04	0.03	0.01	0.00

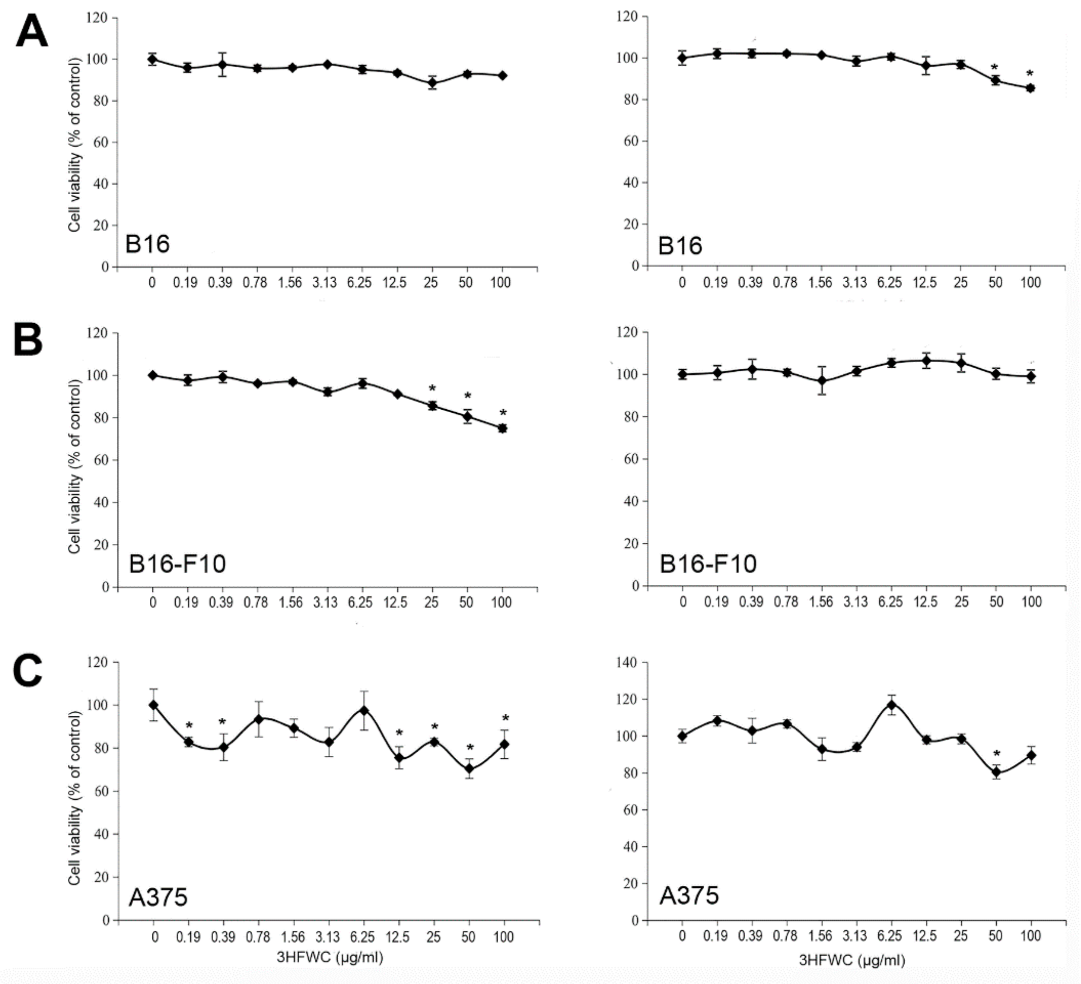

Figure S2. Effect of 3HFWC on melanoma cell viability *in vitro*. (**A**) B16, (**B**) B16-F10, and (**C**) A375 cells were treated with the indicated dose range of 3HFWC (measured as µg/mL of fullerol). The viability was evaluated by MTT (left panel) and crystal violet (CV) (right panel) assays. The results after 48 h (**A**,**B**) and after 72 h incubation (**C**) are shown. *Significant if $p<0.05$ in comparison to untreated cells.

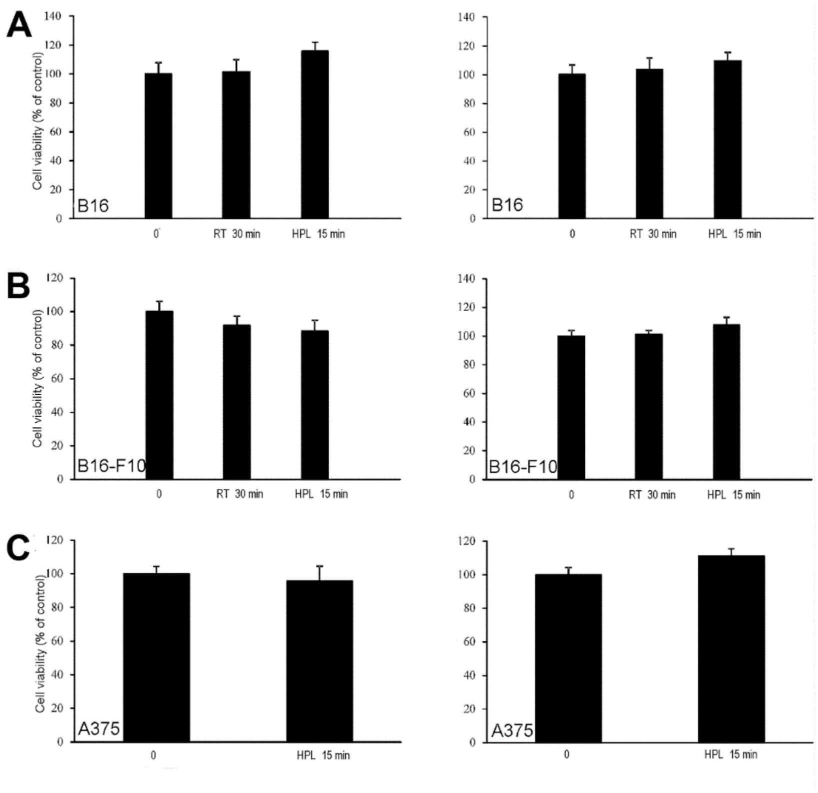

Figure S3. Effect of HPL on melanoma cell viability *in vitro*. (**A**) B16, (**B**) B16-F10, and (**C**) A375 cells were irradiated with HPL for 15 min, or were left at room temperature and light (RT) for 30 min. Nonirradiated cells served as control. The viability was evaluated by MTT (left panel) and crystal violet (CV) (right panel) assays. The results after 48 h (**A**,**B**) and after 72 h incubation (**C**) are shown. * Significant if $p<0.05$ in comparison to untreated cells.

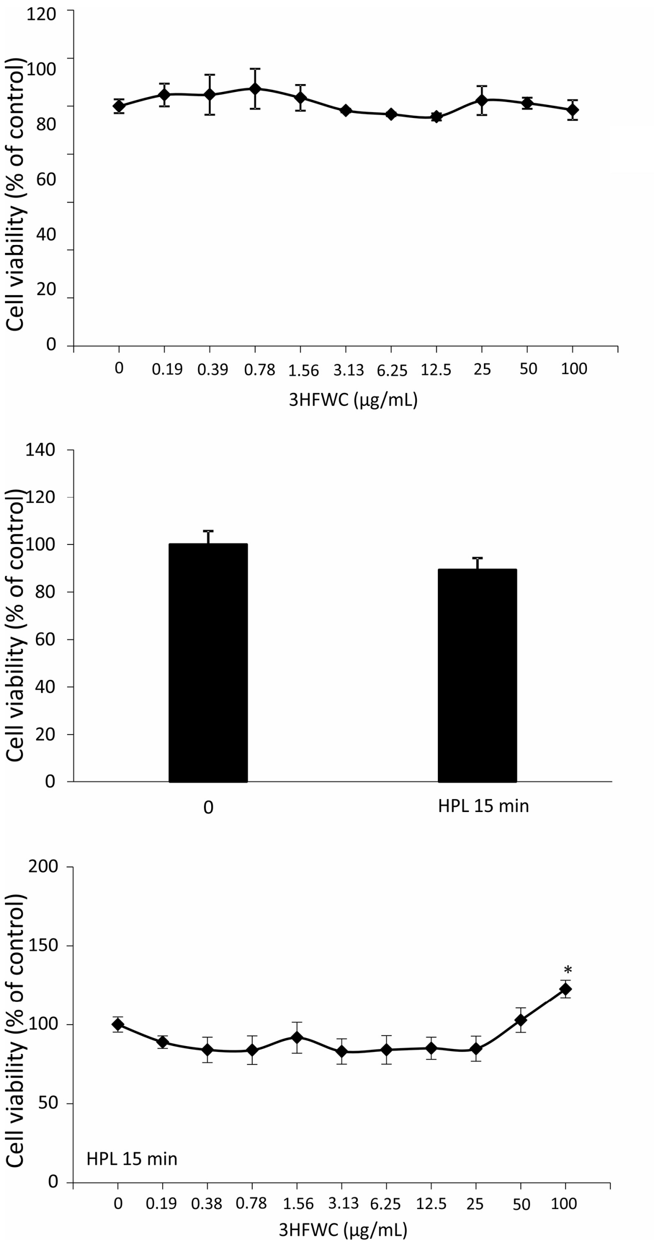

Figure S4. Viability of peritoneal exudate cells (PECs) upon exposure to 3HFWC and/or HPL *in vitro.* Viability of PECs treated with the indicated dose range of 3HFWC (measured as µg/mL of fullerol) alone (upper) or in combination with 15 min HPL irradiation (lower). Viability of PECs treated with HPL for 15 min (middle). The viability was evaluated by CV assay. The results after 48 h incubation are shown. *Significant if $p<0.05$ in comparison to cells untreated with 3HFWC.

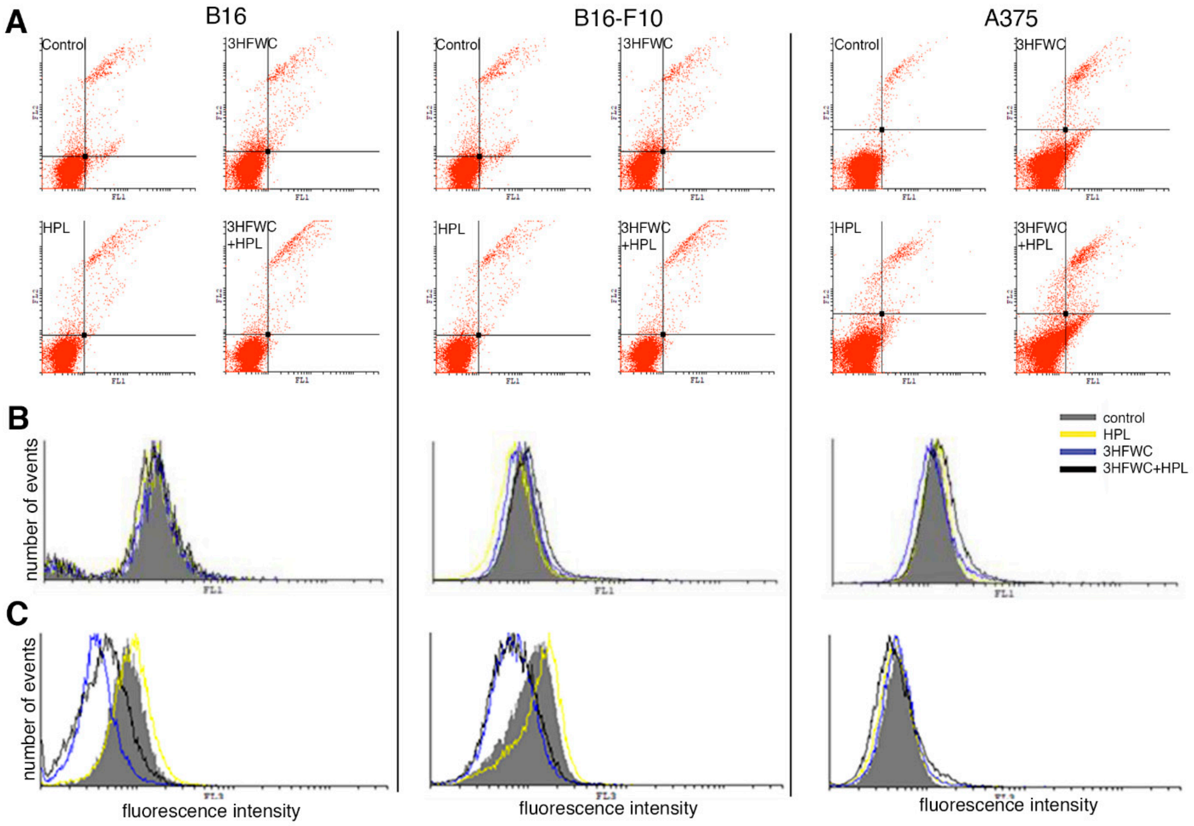

Figure S5. Analysis of cell death induction of melanoma cells treated with 3HFWC and/or HPL. (**A**) Ann V-FITC/PI staining; (**B**) Apostat detection of caspase activity; (**C**) Acridine orange staining of autophagosomes.

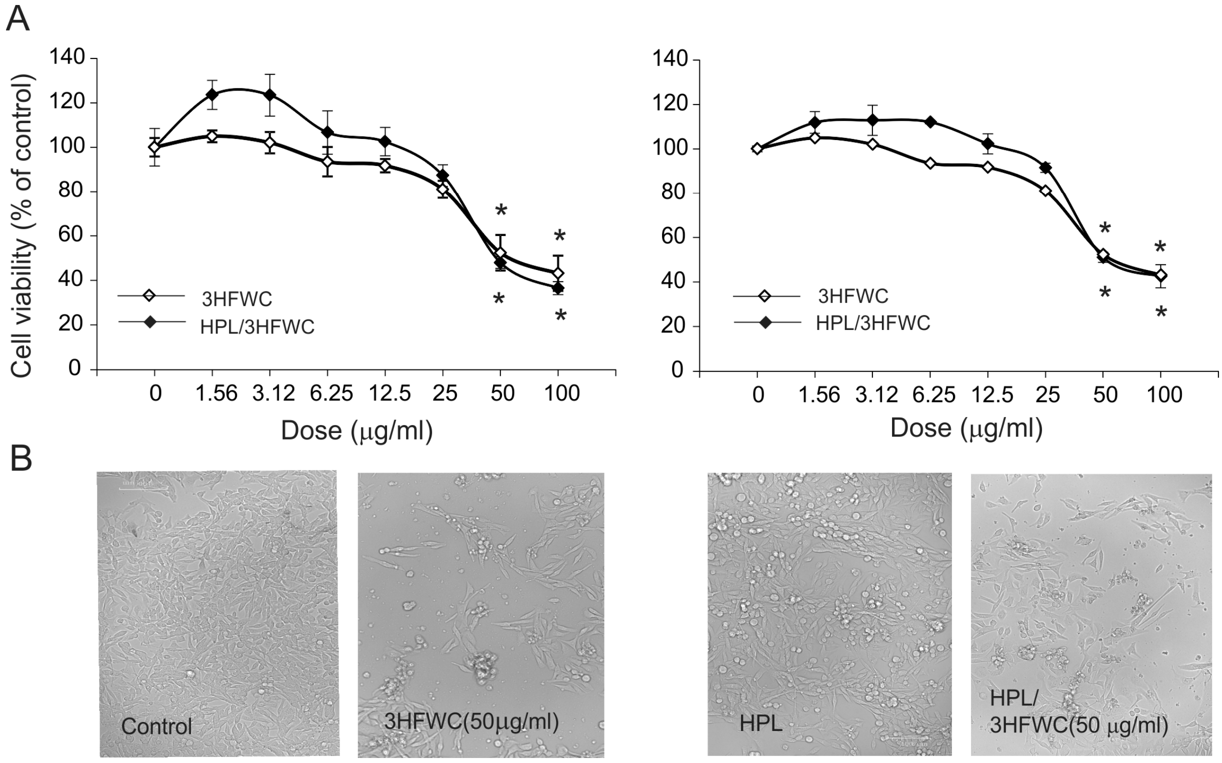

Figure S6. Effect of prolonged exposure to 3HFWC/HPL on B16 melanoma cells viability. B16 cells were treated with the indicated dose range of 3HFWC (measured as μg/mL of fullerol) and irradiated with HPL as described. (**A**) The viability was evaluated by MTT (left panel) and crystal violet (CV) (right panel) assays. (**B**) Representative micrographs of treated cells. The results after 96 h of cultivation were shown. * Significant if $p<0.05$ in comparison to untreated cells.

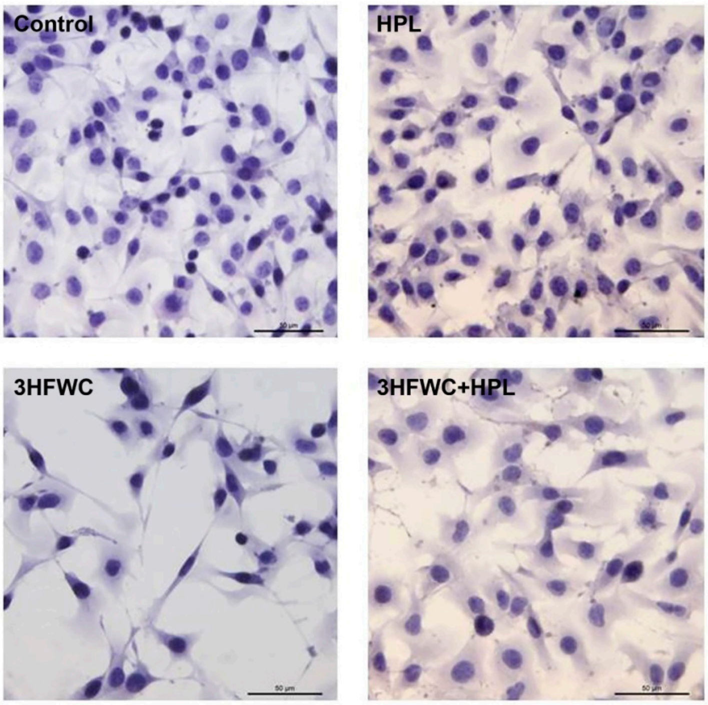

Figure S7. Immunocytochemical analysis of MBP, a Schwann cells marker in B16 melanoma cells treated with 3HFWC and/or HPL. The lack of immunoexpression of MBP indicates that melanoma cells did not differentiate into Schwann-like phenotype after the 3HFWC and/or HPL treatments. Magnification and scale bar: ×400, 50 μm.

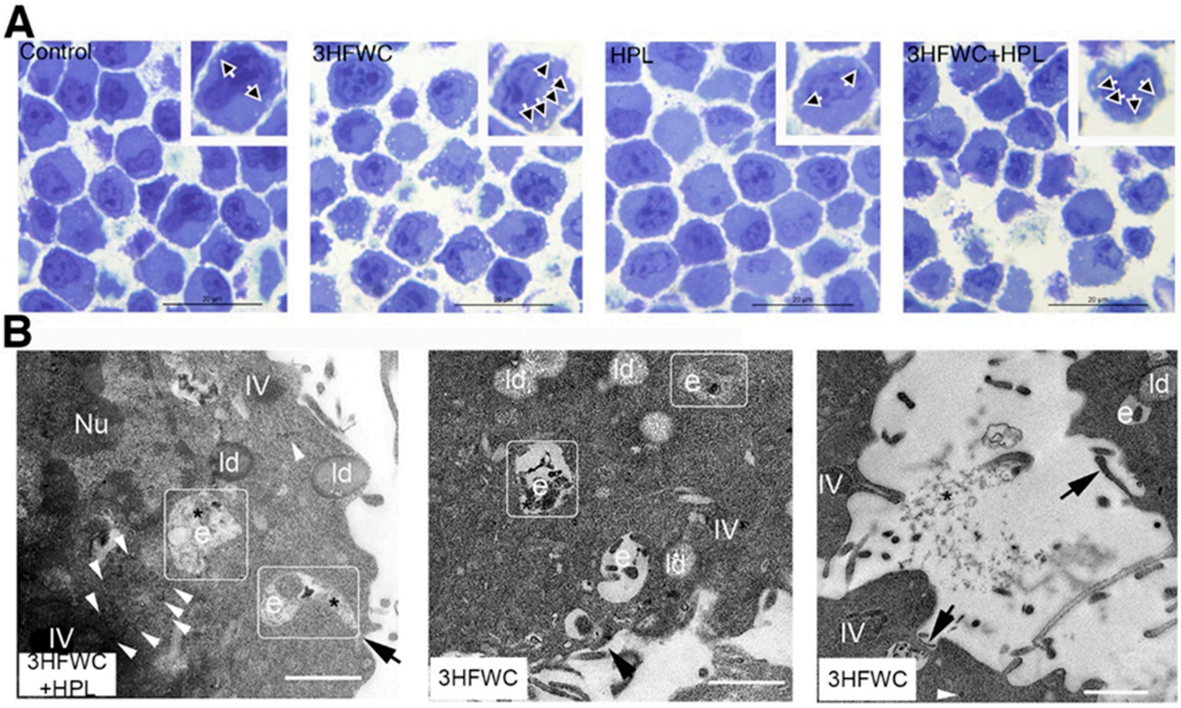

Figure S8. Endocytotic activity of B16-F10 melanoma cells treated with 3HFWC. (**A**) Light microscopic analysis of toluidine blue stained, resin embedded cells has demonstrated increased incidence of endocytotic vesicles (arrows) in 3HFWC- and 3HFWC+HPL-treated cells in comparison to control (untreated) cells. (**B**) Electron-microscopic analysis demonstrated endocytosis of 3HFWC-like granular material (asterisks) after 3HFWC treatments with numerous endosomes (e) visible in these cells. The main mechanism of endocytosis was macropinocytosis (black arrows). Nu – nucleus; ld – lipid droplet; III and IV – pigmented melanosomes (stage III and IV); white arrowheads – clathrin vesicles. Magnification and scale bars: (**A**) x400, 50 μm, (**B**) x13000, 1 μm.
